# Mitochondrial redox impairment and enhanced autophagy in peripheral blood mononuclear cells from type 1 diabetic patients

**DOI:** 10.1016/j.redox.2022.102551

**Published:** 2022-11-24

**Authors:** F. Canet, P. Díaz-Pozo, C. Luna-Marco, M. Fernandez-Reyes, T. Vezza, M. Marti, J.D. Salazar, I. Roldan, C. Morillas, S. Rovira-Llopis, M. Rocha, V.M. Víctor

**Affiliations:** a Service of Endocrinology and Nutrition, University Hospital Doctor Peset, Foundation for the Promotion of Health and Biomedical Research in the Valencian Region (FISABIO), Valencia, Spain; b CIBERehd - Department of Pharmacology, University of Valencia, Valencia, Spain; c Foundation for the Promotion of Health and Biomedical Research in the Valencian Region (FISABIO), Valencia, Spain; d Department of Physiology, School of Medicine, University of Valencia and Institute of Health Research INCLIVA, Valencia, Spain

**Keywords:** Diabetes, Mitochondrial respiration, Oxidative stress, ROS, Autophagy, PBMC

## Abstract

Type 1 diabetes (T1D) involves critical metabolic disturbances that contribute to an increased cardiovascular risk. Leukocytes are key players in the onset of atherosclerosis due to their interaction with the endothelium. However, whether mitochondrial redox impairment, altered bioenergetics and abnormal autophagy in leukocytes contribute to T1D physiopathology is unclear.

In this study we aimed to evaluate the bioenergetic and redox state of peripheral blood mononuclear cells (PBMCs) from T1D patients in comparison to those from healthy subjects, and to assess autophagy induction and leukocyte-endothelial interactions.

T1D patients presented lower levels of fast-acting and total antioxidants in their blood, and their leukocytes produced higher amounts of total reactive oxygen species (ROS) and superoxide radical with respect to controls. Basal and ATP-linked respiration were similar in PBMCs from T1D and controls, but T1D PBMCs exhibited reduced spare respiratory capacity and a tendency toward decreased maximal respiration and reduced non-mitochondrial respiration, compared to controls. The autophagy markers P-AMPK, Beclin-1 and LC3-II/LC3-I were increased, while P62 and NBR1 were decreased in T1D PBMCs versus those from controls. Leukocytes from T1D patients displayed lower rolling velocity, higher rolling flux and more adhesion to the endothelium versus controls.

Our findings show that T1D impairs mitochondrial function and promotes oxidative stress and autophagy in leukocytes, and suggest that these mechanisms contribute to an increased risk of atherosclerosis by augmenting leukocyte-endothelial interactions.

## Highlights

• Leukocytes are sensors of metabolic stress and act as bioenergetic biomarkers.
• Leukocytes from T1D patients produced higher total ROS and superoxide than controls.
• T1D patients had lower levels of fast-acting and total antioxidants in blood.
• Leukocytes from T1D patients showed reduced mitochondrial spare respiratory capacity.
• Autophagy markers in T1D leukocytes show a classic pattern of autophagy activation.

## Graphical abstract

**Figure:**
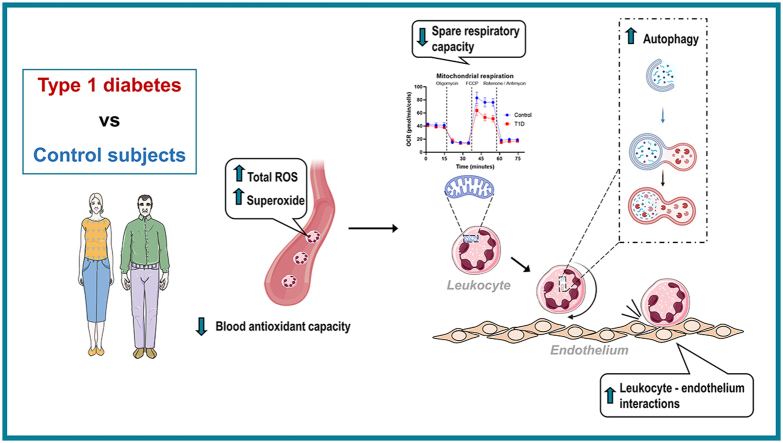
Overview of the study. Partially created with BioRender.com.

## 1 Introduction

Type 1 diabetes (T1D) is an autoimmune disease characterized by insulin deficiency due to pancreatic beta cell destruction, which leads to hyperglycaemia [1].

T cells, classically described as the main mediators of beta cell destruction in T1D [2], are the most abundant cell type in peripheral blood mononuclear cells (PBMCs), making up approximately 45–70% [3]. The study of PBMCs provides important insight into the immunological status of patients with T1D [4], and circulating leukocytes are sensors of metabolic stress in patients and act as bioenergetic biomarkers [5]. Importantly, mitochondrial dysfunction in PBMCs is known to reflect the systemic inflammation and oxidative stress that underlie cardiovascular diseases [6].

The risk of cardiovascular disease among T1D patients is well established, and cardiovascular disease is the co-morbidity responsible for the greatest proportion of deaths in T1D patients [7]. The major mechanisms underlying hyperglycaemia-induced vascular damage are activated by mitochondrial overproduction of reactive oxygen species (ROS) [8]. Superoxide is the initial ROS molecule formed by the mitochondrial electron-transport chain and is then converted to other reactive species that can damage cells in several ways [8]. ROS are one of the major inducers of autophagy [9]. Physiological ROS levels maintain cellular homeostasis and autophagy supports this balance. However, under oxidative stress conditions autophagy is triggered and acts as a critical mediator of pathological responses to excessive ROS production [10,11].

Given the critical role of peripheral circulating cells as sensors of metabolic stress and their potential involvement in the vascular complications of T1D, we aimed to determine the bioenergetic and redox state of leukocytes, their interaction with the endothelium, and autophagy induction in T1D patients and to compare these parameters with those in healthy subjects.

## 2 Material and methods

### 2.1 Study population

Fifty-two healthy volunteers and forty-four patients with T1D were recruited from the Endocrinology and Nutrition Service of University Hospital Dr. Peset, in Valencia, Spain and were matched according to age and sex. Participants were informed of the details of the study and gave their written consent. The Ethics Committee of Clinical Investigation of the University Hospital Dr. Peset reviewed and approved the study protocol (ID: 98/19). The study was conducted under the ethical principles of the Helsinki Declaration.

Exclusion criteria were any documented history of cardiovascular disease (ischaemic cardiopathy, stroke, peripheral arteriopathy or any cardiovascular diseases associated with cardiovascular risk); and any severe inflammatory, infectious, or autoimmune disease.

### 2.2 Physical and biochemical determinations

Patients underwent a physical examination to determine weight, height, waist circumference, systolic (SBP) and diastolic (DBP) blood pressure. After 12 h of fasting, blood samples were collected and biochemical determinations were carried out in our hospital's Clinical Analysis Service, as previously described [12].

### 2.3 Leukocyte extraction

Neutrophils and PBMCs were isolated from blood collected in EDTA tubes as described previously [13]. For neutrophils, 1.0 × 10^6^ cells were resuspended in RPMI media supplemented with 10% v/v FBS (Biowest) and used for leukocyte-endothelium interaction assays. In the case of PBMCs, an aliquot of 2.0 × 10^6^ cells was used for real-time metabolic flux analysis and the remaining cells were stored at −80 °C for subsequent western blotting.

### 2.4 Leukocyte-endothelium interaction assays

To mimic the interaction between leukocytes and the endothelial cell layer inside the blood vessel and thus simulate physiological conditions, we used an *ex vivo* model based on a parallel plate flow chamber as described before [14]. In short, a suspension of leukocytes (1.0 × 10^6^ cells/mL) was perfused over a monolayer of primary human umbilical vein endothelial cells (HUVECs) at a flow rate of 0.36 ml/min. Real time images of the flow-exposed monolayer were recorded for 5 min and analysed. Leukocyte rolling flux, rolling velocity and adhesion were determined as described elsewhere [15].

### 2.5 Measurement of blood antioxidant capacity

Blood antioxidant capacity was measured using e-BQC Lab device (Bioquochem) according to the manufacturer's protocol. This electrochemical method measures the total antioxidant capacity of blood in micro-Coulombs (μC) and distinguishes between fast- and slow-acting antioxidants.

### 2.6 Flow cytometry assay

The whole blood of patients (500 μl) was processed as previously described [13]. The fluorochromes (Invitrogen) 2′,7′-Dichlorodihydrofluorescein diacetate (5 μM, DCFH) and Hydroethidine (5 μM, HE) were employed to measure total free radicals and superoxide content, respectively. 10,000 cells were analysed in each experiment.

### 2.7 Real-time metabolic flux analysis

The mitochondrial function of PBMCs was measured in real-time with a Seahorse XFp analyzer (Agilent) using the XFp Cell Mito Stress Test Kit (Agilent) and following the manufacturer's instructions.

Immediately after extraction of PBMCs, the pellets were resuspended with Seahorse XF DMEM medium pH 7.4 (Agilent) containing 1 mM pyruvate, 2 mM glutamine and 10 mM glucose and seeded on culture miniplates that had been pretreated with Poly-d-Lysine (0.1 mg/ml) at a density of 3.5 × 10^5^ cells/well in 180 μL. Compounds were loaded into the cartridge injection ports following the manufacturer's volume recommendations, such that the final concentration in each well was Oligomycin A 1.5 μM, FCCP 1.0 μM and Rotenone/Antimycin 1 μM.

### 2.8 Protein expression analysis of autophagy markers

PBMC protein extraction, quantification and Western blot were carried out as described previously [12,16]. The details of primary antibodies for AMPK-P, Beclin 1, P62, NBR1, LC3A/B and β-Actin are in Supplementary Table 1.

### 2.9 Statistical analysis

R version 4.1.2, RStudio IDE (www.rstudio.com) and the Tidyverse package [17] were employed for data management and visualization. GraphPad Prism version 8.02 (GraphPad Software, www.graphpad.com) was used for statistical analysis and data presentation. Statistical significance (P-value <0.05) between two groups was assessed by an unpaired T test for normally distributed variables and Mann–Whitney *U* test for non-normally distributed variables. For correlation analysis we calculated Spearman's correlation coefficient and applied LOESS regression.

## 3 Results

### 3.1 Anthropometrical parameters and biochemical determinations

Table 1 shows the anthropometric and biochemical data of the fifty-two healthy controls and forty-four T1D patients. There was no difference in gender distribution between the groups, with 62% and 55% women in the control and T1D groups, respectively. The mean age was 40.4 years among controls and 43.6 years in the T1D group, and the mean duration of diabetes in the latter group was 15.7 years. There were no significant differences in weight, waist circumference, body mass index (BMI) or blood pressure between groups.

**Table 1:** Anthropometric characteristics, biochemical determinations, and pharmacological treatment of the study population.

Characteristics	Control	Type 1 diabetes	p-value
n	52	44	
Sex (% women)	62%	55%	ns
Age (years)	40.4 ± 11.5	43.6 ± 12.3	ns
Duration of diabetes (years)	–	15.7 ± 8.7	
Weight (kg)	75.4 ± 16.3	73.9 ± 13.3	ns
Waist circumference (cm)	85.1 ± 13.0	87.8 ± 13.8	ns
BMI (kg/m^2^)	25.9 ± 4.8	25.7 ± 4.4	ns
SBP (mm Hg)	118 ± 13	125 ± 20	ns
DBP (mm Hg)	73 ± 9	74 ± 10	ns
Glucose (mg/dL)	88.0 (83.75–96.0)	133.0 (112.5–232.0)	<0.001
HbA1c-DCCT (%)	5.2 ± 0.3	7.6 ± 1.0	<0.001
Total cholesterol (mg/dL)	185.6 ± 31.1	160.4 ± 26.7	<0.001
HDL-c (mg/dL)	57.1 ± 11.9	57.7 ± 14.6	ns
LDL-c (mg/dL)	112.1 ± 25.7	86.2 ± 23.0	<0.001
Triglycerides (mg/dL)	71.5 (53.8–104.3)	71.0 (55.5–92.5)	ns
hs-CRP (mg/L)	0.95 (0.4–2.25)	1.5 (0.8–3.6)	ns

**Treatment**		
*Non-insulin antidiabetic drugs*		13.6%
Metformin	–	2.3%
Metformin + SGLT2 inhibitors	–	4.5%
Metformin + DPP4 inhibitors	–	2.3%
SGLT2 inhibitors		4.5%
*Lipid-lowering medication*		56.8%
Statins	–	34.1%
Statins + Fibrates	–	4.5%
Statins + Ezetimibe	–	18.2%
*Antihypertensive medication*	–	13.6%

Data are expressed as mean ± SD for normally distributed variables or median and interquartile range for non-normally distributed data. A Chi-square test was performed to determine differences in the proportion of sexes between groups. An unpaired T test or Mann Whitney *U* Test was performed to determine differences between groups. Abbreviations: BMI: body mass index, DBP: diastolic blood pressure, HbA1c: glycated hemoglobin, HDL-c: high-density lipoprotein cholesterol, hs-CRP: high-sensitive C-reactive protein, LDL-c: low-density lipoprotein cholesterol; ns: not significant, SBP: systolic blood pressure.

As expected, the T1D group displayed higher fasting glucose concentrations and HbA1c than controls. T1D patients presented lower levels of total cholesterol and LDL cholesterol, which may have been due to the lipid-lowering medication that 57% of them were taking.

### 3.2 ROS production and antioxidant capacity

**Fig. 1:**
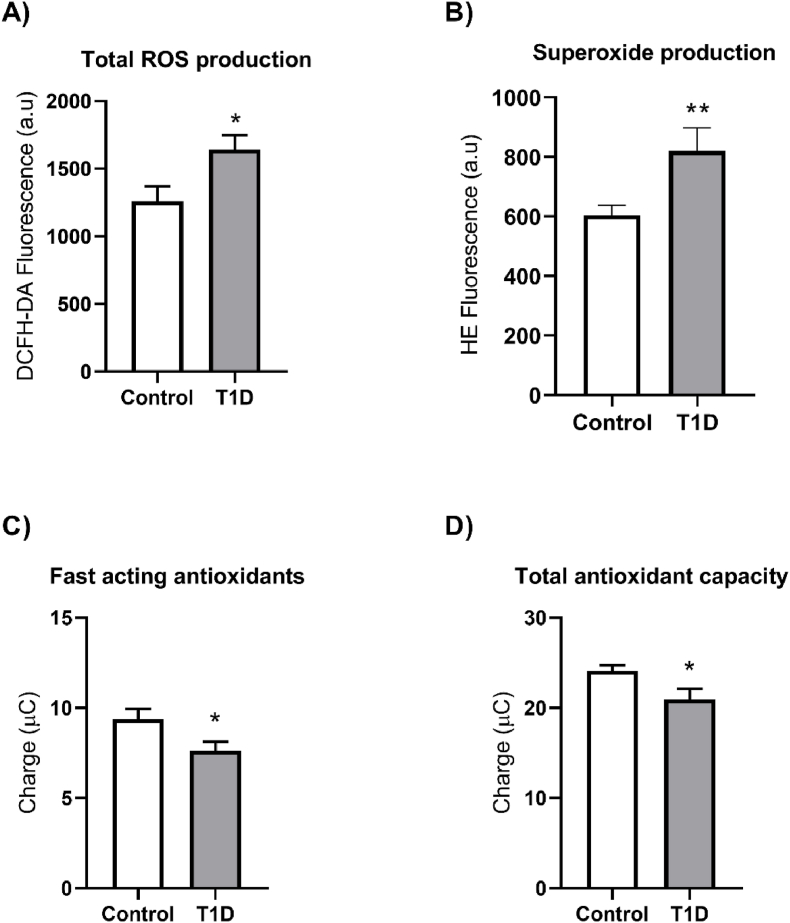
Leukocyte reactive oxygen species production and blood antioxidant capacity in control and type 1 diabetic patients. A) Total ROS production measured by flow cytometry as DCFH-DA fluorescence intensity (a.u), B) Superoxide production measured by flow cytometry as HE fluorescence intensity (a.u), C) Fast acting antioxidant measurement in blood (μC) and D) Blood total antioxidant capacity (μC), measured by an electrochemistry method. Values in the bar charts represent mean ± SEM. At least 11 samples for each group were included. Comparisons were made using a Mann-Whitney test for Total ROS production and using a *t*-test for Superoxide production, Fast acting antioxidants, and Total antioxidant capacity. *p < 0.05, **p < 0.01. Abbreviations: a.u.: arbitrary units, DCFH-DA: 2′,7′-Dichlorofluorescin diacetate, HE: Hydroethidine, ROS: reactive oxygen species, T1D: type 1 diabetes.

Hyperglycaemia contributes to the production of large amounts of ROS by mitochondrial and non-mitochondrial sources, which are associated with the development of cardiovascular complications [18,19]. Leukocytes from T1D patients produced higher amounts of total ROS (Fig. 1A, p < 0.05) and the superoxide radical (Fig. 1B, p < 0.01) than those from controls. In addition, we measured blood antioxidant capacity as a reflection of systemic oxidative stress and found that T1D patients had lower levels of fast-acting and total antioxidants (Fig. 1C–D, both p < 0.05).

### 3.3 Measurement of mitochondrial function

**Fig. 2:**
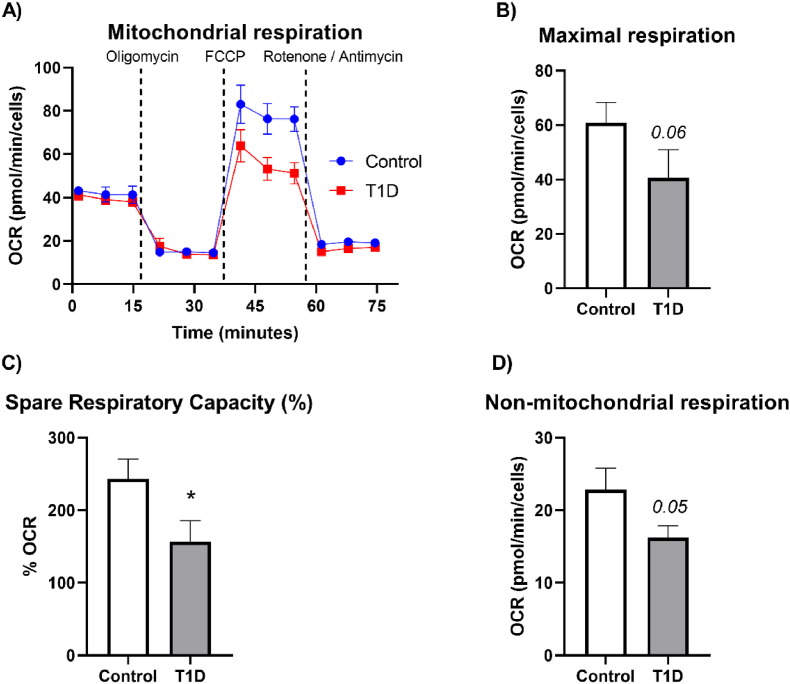
Mitochondrial function on leukocytes from control and type 1 diabetic patients. A) Representative OCR during Mito Stress Test, B) Maximal respiration is the maximum rate of respiration that the cell can achieve, C) Spare respiratory capacity (%) indicates the capability of the cells to adapt to an energy demand, expressed as percentage of basal respiration and D) Non-mitochondrial respiration, indicating how much oxygen is still consumed by a subset of cellular enzymes after the injection of rotenone and antimycin A. Values in the bar charts represent mean ± SEM of at least 7 independent experiments. Comparisons were made using a *t*-test. *p < 0.05. Abbreviations: OCR: Oxygen consumption rate, T1D: type 1 diabetes.

**Supplementary Fig. 1:**
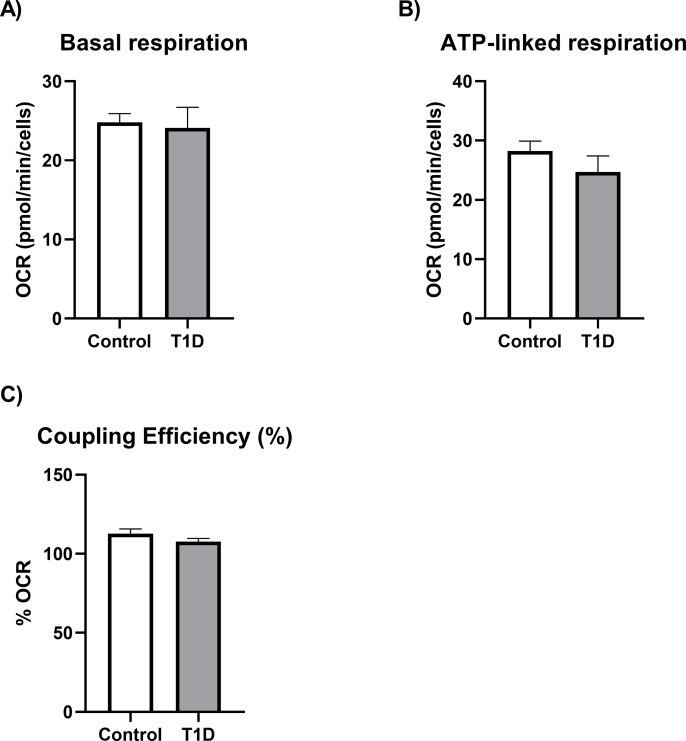
Mitochondrial function on leukocytes from control and type 1 diabetic patients. A) Basal respiration shows energetic demand of the cell under baseline conditions, B) ATP-linked respiration shows ATP produced by the mitochondria that contributes to meeting the energetic needs of the cell, and C) Coupling efficiency (%) is the proportion of oxygen consumed to drive ATP synthesis compared to oxygen consumed during proton leak. Values in the bar charts represent mean ± SEM of at least 7 independent experiments. Measurements of the OCR were done during the Mito Stress Test. Comparisons were made using a *t*-test. Abbreviations: OCR: Oxygen consumption rate, T1D: type 1 diabetes.

Analysis of oxygen consumption rate (OCR) during the Mito Stress test in T1D vs control subjects (Fig. 2A) revealed similar basal and ATP-linked respiration in both groups (Supplementary Figs. 1A–B). Interestingly, leukocytes from T1D patients displayed reduced maximal respiration rate (p = 0.06), spare capacity (p < 0.05) and non-mitochondrial respiration (p = 0.05), compared to controls (Fig. 2B–D). There were no differences in mitochondrial coupling efficiency (Supplementary Fig. 1C).

### 3.4 Autophagy marker expression

**Fig. 3:**
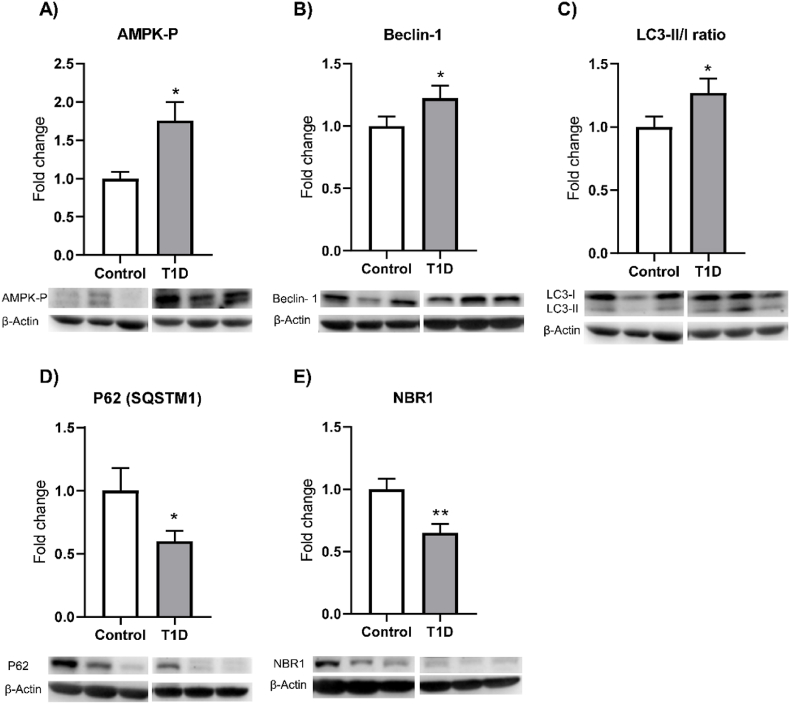
Expression of autophagy markers on leukocytes from control and type 1 diabetic patients. Protein levels of autophagy markers relative to β-Actin signal A) AMPK-P, B) Beclin-1, C) LC3-II/I ratio. D) P62 (SQSTM1) and E) NBR1; expressed as fold-change with respect to the control group. Representative images of the Western blot experiments are shown for each assessed protein. Values in the bar charts represent mean ± SEM. Quantification of Beclin-1, P62, NBR1 and LC3-II/I ratio was performed using at least 15 samples for each group, and at least 8 samples for each group were used for quantification of AMPK-P. Images show representative western blots including 3 biological replicates. Comparisons were made using a *t*-test. *p < 0.05, **p < 0.01. Abbreviations: T1D: type 1 diabetes.

Given that autophagy is a major defence against oxidative stress, we next determined whether autophagic flux was activated in the leukocytes of our T1D patients. We observed increased protein expression of P-AMPK and Beclin-1 (Fig. 3A–B, both p < 0.05), both markers of autophagy induction. Moreover, leukocytes from T1D displayed increased conversion of LC3-I to LC3-II (Fig. 3C, p < 0.05) and decreased amounts of the cargo proteins P62 (Fig. 3D, p < 0.05) and NBR1 (Fig. 3E, p < 0.01), all indicative of autophagosome maturation.

**Supplementary Fig. 2:**
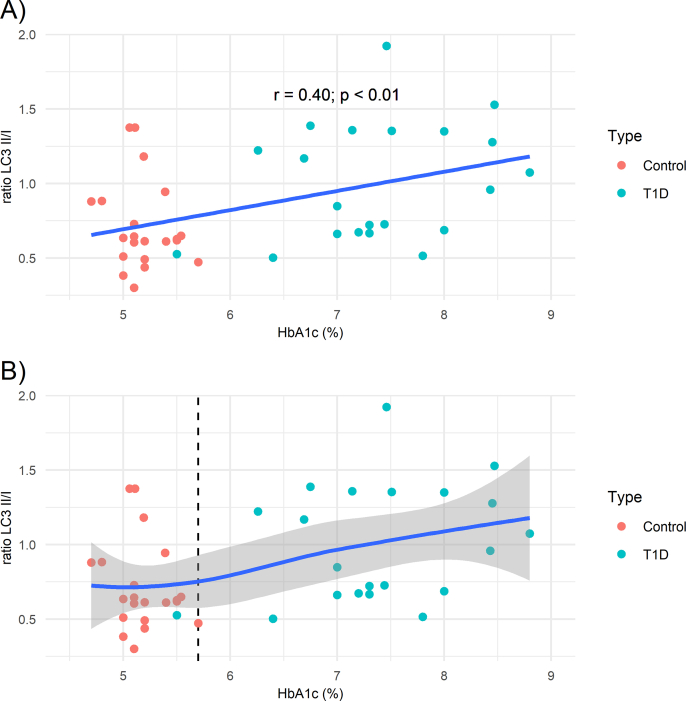
Correlation plots between HbA1c (%) levels and leukocyte expression of LC3-II/I ratio on control and type 1 diabetic patients. A) Linear regression (blue line), Spearman's correlation coefficient (r) and p value. B) Trend line (blue) obtained with the LOESS regression algorithm (span = 1.0). The grey area shows the 95% confidence interval of the regression. The dashed line intercepts the x-axis on HbA1c (%) = 5.7, the upper limit of normal HbA1c (%) levels. Abbreviations: LOESS: Locally Weighted Smoothing, T1D: type 1 diabetes.

Interestingly, we found a positive correlation between HbA1c (%) and the LC3-II/I ratio (r = 0.40, p < 0.01, Supplementary Fig. 2A), and a tendency for said ratio to increase when HbA1c exceeded 5.7% (Supplementary Fig. 2B).

### 3.5 Leukocyte-endothelium interactions

**Fig. 4:**
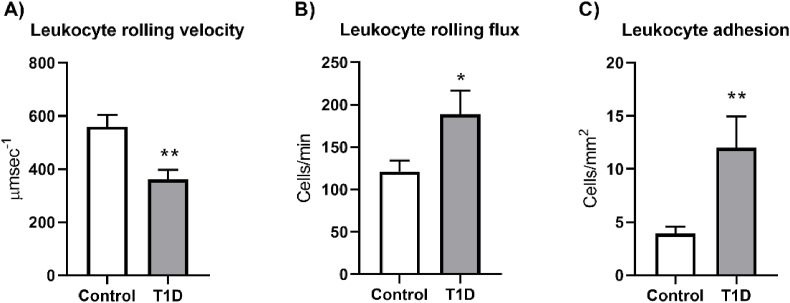
Leukocyte-endothelium interaction parameters in control and type 1 diabetic patients. A) Mean velocity of rolling leukocytes across the endothelial cell layer (μm/sec), B) Number of leukocytes rolling across the endothelial cell layer per unit of time (cells/min) and C) Number of leukocytes attached to 1 mm^2^ of endothelial cell layer (cell/mm^2^). Values in the bar charts represent mean ± SEM. Parameters were measured using an *ex vivo* model based on a parallel plate flow chamber and using at least 12 samples for each group. Comparisons were made using a Mann-Whitney test for rolling velocity and a *t*-test for rolling flux and adhesion. *p < 0.05, **p < 0.01.

The adhesion of leukocytes to the endothelium is a hallmark of the inflammatory process at the onset of atherosclerosis. In the present study we found that T1D patients presented increased leukocyte-endothelium interactions, as their leukocytes displayed reduced rolling velocity (Fig. 4A, p < 0.01) and higher numbers of their leukocytes were observed to roll (Fig. 4B, p < 0.05) or attach to the endothelium and remain stationary (Fig. 4C, p < 0.01) in comparison to those of control subjects.

## 4 Discussion

T1D is a serious chronic illness usually diagnosed at a young age. T1D patients depend on exogenous insulin throughout life, and their wellbeing is typically compromised by both hyperglycaemia (a consequence of poor control of the disease) and hypoglycaemia, due to improper insulin treatment [1].

Chronic hyperglycaemia results in the overloading of the mitochondrial electron transport chain, which increases ROS production and undermines mitochondrial function. In T1D subjects, we have observed decreased blood antioxidant capacity with respect to controls, as well as higher leukocyte total ROS and superoxide production. In line with our findings, Devaraj et al. observed higher superoxide anion levels in monocytes from T1D patients compared with controls [20]. In addition, we observed that mitochondria in leukocytes from T1D patients displayed a reduced spare respiratory capacity. Spare capacity estimates mitochondrial health and flexibility, indicating the cell's bioenergetic adaptation to pathological stress. Inadequate spare respiratory capacity is associated with cardiovascular and neurological chronic diseases [21], and with the development of diabetic complications [22,23]. In this context, Czajka et al. compared PBMCs from diabetic patients with and without nephropathy; while they found similar basal and ATP-linked respiration in both groups, they detected metabolic inflexibility in the former, manifested as reduced maximal and spare respiratory capacity, [22]. In our T1D cohort, we observed similar bioenergetic maladaptation, even in the absence of nephropathy. With regard to this discrepancy, the nephropathy group in the Czajka et al. study included a large proportion of type 2 diabetic patients, and so additional factors, such as insulin resistance, could have been driving the mitochondrial impairment observed.

Together with mitochondrial dysfunction and excessive ROS, we have witnessed a classic pattern of autophagy activation, with low p62 and NBR1 protein levels and increased levels of Beclin1 and the LC3-II isoform. Interestingly, P-AMPK levels were also increased in PBMCs of T1D patients. In this sense, AMPK may be a protein marker of autophagy induction upon mitochondrial ROS production [9], as it becomes activated upon exposure to H_2_O_2_, a process that occurs through oxidative modifications (including *S*-glutathionylation of the AMPKα and AMPKβ subunits) [9,24]. Moreover, modifications of Atg4 by H_2_O_2_ have been found to inactivate its hydrolysing activity towards LC3, which allows accumulation of the pro-autophagic LC3-II isoform [9,25].

Furthermore, our data show a positive correlation between HbA1c and LC3-II, supporting an association between increased autophagy and poor glycaemic control, and might be an indication of excessive cellular stress due to chronic hyperglycaemia.

There is a crosstalk between increased ROS production, mitochondrial dysfunction, inflammation, and autophagy. In obesity, it has been observed that autophagy is upregulated due to over activated pro-inflammatory signals [26]. Upregulation of autophagy by TNFα is induced by ROS production and is mediated by activation of the Jun kinase pathway and inhibition of the Akt pathway [[26], [27], [28]]. Accordingly, we have previously observed increased levels of serum TNFα in T1D patients [29]. Moreover, it has been shown in animal studies that the proinflammatory transcription factor NF-κB is involved in the induction of autophagy in response to ROS [26,30].

Lastly, and in accordance with a previous analysis performed by our group in a different cohort of T1D patients [29], we demonstrate that leukocyte-endothelial interactions are exacerbated in the diabetic population. In support of this proinflammatory state, other authors have shown that monocytes isolated directly from the blood of T1D patients secrete pro-inflammatory cytokines such as IL-1β and IL-6 [31]. In relation to this, we and other groups have previously demonstrated an increase in circulating adhesion molecules in T1D, which could be the reason why leukocytes are attracted to the endothelial walls [20,29].

## 5 Conclusions

Inflammation and oxidative stress are key mechanisms contributing to atherosclerosis, and leukocytes play a pivotal role in this process. In this study, we have characterized the phenotype of leukocytes from T1D patients, and this phenotype includes augmented ROS, impaired mitochondrial respiration rate and activated autophagy. These alterations occur in parallel to an overall reduction in antioxidant defences and increased interaction between leukocytes and endothelial cells. These findings support the idea that T1D impairs mitochondrial function and promotes oxidative stress and autophagy in leukocytes, and that these mechanisms contribute to an increased risk of atherosclerosis by augmenting leukocyte-endothelial interactions. Further studies are needed to confirm the directionality of these processes.

## Funding

This study was financed by Carlos III Health Institute and by the European Regional Development Fund (ERDF ‘‘A way to build Europe’‘) [PI22/00424, PI19/00838, PI19/0437], CIBERehd [CB06/04/0071] and Ministry of Health of the Valencian Regional Government [PROMETEO/2019/027, ACIF/2020/370 (P.D-P), CIAPOS/2021/142 (T.V.), GRISOLIAP/2019/091(F.C.)]. S.R-L is recipient of a Maria Zambrano fellowship [ZA21-049] from the requalification of the Spanish university system from the Ministry of Universities of the Government of Spain, financed by the European Union, NextGeneration EU. C.L-M was supported by Erasmus+ internship grant through Uppsala University, Sweden.

## Declaration of competing interest

The authors have no competing interest to declare.

## Data Availability

Data will be made available on request.
