# Supplementary material for: Mitochondrial redox impairment and enhanced autophagy in peripheral blood mononuclear cells from type 1 diabetic patients
Source: Redox Biol. 2022 Nov 24;58:102551. doi: 10.1016/j.redox.2022.102551 (PMC9713367; doi:10.1016/j.redox.2022.102551)
Supplement: Multimedia component 1 [file mmc1.docx]

**Supplementary table 1:** Antibodies used for Western blotting

| **Primary antibodies** | | | | | | | | | | |
| --- | --- | --- | --- | --- | --- | --- | --- | --- | --- | --- |
| **Targets** | **Clonality** | **Brand** | **Reference** | | | **Dilution** | | **Host** | | **Blocking buffer (in TBS-T)** |
| AMPK-P | Monoclonal | Abcam | Ab133448 | | | 1:1000 | | Rabbit | | BSA 5% |
| SQSTM/P62 | Monoclonal | Abnova | H00008878-M01 | | | 1:1000 | | Mouse | | Milk 5% |
| LC3A/B | Polyclonal | Cell Signaling | 4108S | | | 1:1000 | | Rabbit | | BSA 5% |
| Beclin 1 | Monoclonal | Abcam | Ab207612 | | | 1:1000 | | Rabbit | | Milk 5% |
| β-Actin | Polyclonal | Sigma-Aldrich | A5060 | | | 1:2000 | | Rabbit | | Milk 5% |
| **Secondary antibodies** | | | | | | | | | | |
| **Target** | **Clonality** | **Brand** | | **Reference** | **Dilution** | | **Host** | | **Blocking buffer (in TBS-T)** | |
| Rabbit | Polyclonal | Vector | | PI-1000 | 1:2000 | | Goat | | Same as primary ab. | |
| Mouse | Polyclonal | Thermo-Fisher | | 31420 | 1:2000 | | Goat | | Same as primary ab. | |
